# Survival Outcome in Critically Ill Patients Receiving Extracorporeal Membrane Oxygenation Support: Early Experience from a University Hospital in Thailand

**DOI:** 10.1055/s-0043-1761444

**Published:** 2023-02-13

**Authors:** Pongsanae Duangpakdee, Sasitorn Sakkarat, Surasak Sangkhathat

**Affiliations:** 1 Department of Surgery, Division of Cardio-Thoracic Surgery, Faculty of Medicine, Prince of Songkla University, Hat Yai, Songkhla, Thailand; 2 Department of Surgery and Translational Medicine Research Center, Faculty of Medicine, Prince of Songkla University, Hat Yai, Songkhla, Thailand

**Keywords:** cardiopulmonary failure, extracorporeal membrane oxygenator, life support

## Abstract

**Objective** Extracorporeal membrane oxygenation (ECMO) is a relatively new technology used for life support in patients with cardiopulmonary failure from various causes. The objective of this study is to review the first 5-year experience in adopting this technology in a teaching hospital in southern Thailand.

**Methods** The data of ECMO-supported patients in Songklanagarind Hospital, from the years 2014 to 2018, were retrospectively reviewed. Data sources were from electronic medical records and the database of the perfusion service. Parameters in focus included prior conditions and indications of ECMO, type of ECMO and cannulation method, complications during and after the treatment, and discharge statuses.

**Results** A total of 83 patients received ECMO life support during the 5-year period and the number of cases per year increased. The proportion of venovenous: venoarterial ECMO in our institute was 49:34 cases and there were three cases who used ECMO as a part of cardiopulmonary resuscitation. Moreover, there were 57 cases who used ECMO for cardiac failure and 26 cases were for respiratory causes, while premature withdrawal was decided in 26 cases (31.3%). Overall survival from ECMO was 35/83 cases (42.2%) and survival to discharge was 32/83 (38.6%). During therapy, ECMO could restore serum pH to the normal range in all cases. Furthermore, those who used ECMO for respiratory failure had significantly higher survival probability (57.7%) when compared to the cardiac counterpart (29.8%, *p* -value = 0.03). Patients with younger ages also had significantly better survival outcomes. The most common complications were cardiac (75 cases, 85.5%), followed by renal (45 cases, 54.2%), and hematologic systems (38 cases, 45.8%). In those who survived to discharge, average ECMO duration was 9.7 days.

**Conclusion** Extracorporeal life support is a technology that bridges the patients with cardiopulmonary failure to their recovery or definitive surgery. Despite the high complication rate, survival can be expected, especially in respiratory failure cases and relatively young patients.

Extracorporeal membrane oxygenation (ECMO) was first described by Bartlett et al in 1977 as a bridge to recovery that provides temporary cardiopulmonary support for critically ill patients in whom conventional treatment options have failed. 1 During typical ECMO support, a patient's whole blood circulates through an extracorporeal circuit containing a centrifugal pump to a membrane oxygenator where gases are exchanged before returning to the patient's circulation. In the venovenous (VV)-ECMO and venoarterial (VA)-ECMO systems, blood passes from the major vein through the oxygenator and the oxygenated blood is returned to the venous and arterial sides, respectively. 2

With the purpose of bridging to recovery, clinical uses of ECMO vary, including temporary support while awaiting physiologic recovery, bridging to surgery or cardiac intervention, waiting for heart and lung transplantation, and waiting for proper decision-making. 3 The primary causes of cardiopulmonary failure that lead to ECMO support include acute respiratory distress syndrome (ARDS), 4 poisoning, 5 severe respiratory failure caused by viral infection, 6 refractory cardiogenic shock, 7 and refractory septic shock. 8 Recently, the uses of ECMO were extended to more situations such as bridging to organ donation in non-heart-beating braindead donors 9 and use as an adjunct to cardiopulmonary resuscitation (ECMO cardiopulmonary resuscitation [ECPR]). 10 With a wide range of clinical indications, the outcomes of ECMO vary greatly, from 25% survival to discharge in resuscitative ECMO to 50% in refractory cardiogenic shock, and 56% in influenza-related ARDS. 11 In addition, ECMO is related to various complications such as hemolysis, coagulopathy, and thromboembolic events.

Songklanagarind Hospital is a tertiary-level university hospital in southern Thailand with a 1,000-bed capacity. ECMO in the hospital was initiated in the year 2014 when our multidisciplinary intensive care team was developed. 12 Until the end of 2018, we have performed extracorporeal life support 83 times in both adults and children with various clinical indications. The aim of this study is to appraise our early experience in performing this procedure, in terms of the clinical settings involved and outcomes, especially the procedural complications we encountered. In addition, we analyzed for factors associated with outcomes in our series. Data from this study might be beneficial for other intensive care units that are setting up this facility.

## Patients and Methods

Medical records of all consecutive cases in whom an ECMO (Maquet Rotaflow and Novalung) was used during their stay in the intensive care facilities in Songklanagarind Hospital, Prince of Songkla University, were reviewed under permission from the Human Research Ethics Committee, Faculty of Medicine, Prince of Songkla University. Case registration was retrieved from the perfusion service database. Data sources were from the electronic medical records (HIS system, Songklanagarind Hospital), perfusionists' records, and radiological materials review.

### Data Collection Method and Variables of Interest

Patient characteristics mean variables related to the patients' underlying conditions and treatment before ECMO support—including arterial blood gas parameters and ventilator settings, primary indications for ECMO, and detail conditions of ECMO (ECMO type, cannulation type, ECMO hours, circuit problem encountered, and complications during the support)—and until 4 weeks post-procedure, were collected. Data were retrieved from the electronic medical records, imaging records, and ICD-9 summaries of diagnosis.

The main indications for ECMO support were broadly categorized as either cardiac or respiratory failure. The cardiac failure group was further subcategorized into cardiogenic shock (acute myocardial infarction, cardiomyopathy, ECPR, pulmonary emboli, and myocarditis), post-cardiotomy syndrome, and cardiac transplantation recipients. In contrast, the respiratory failure subgroup was categorized into refractory ARDS and other causes.

Regarding ECMO modes, the VV mode included routing blood from the venous system (femoral vein) to return to the venous system (femoral vein or internal jugular vein), whereas the VA mode included routing blood from the venous system to return to the arterial system, which can be either peripheral cannulation (i.e. femoral vein to femoral artery) or central cannulation (draining from vena cava/right atrium to aorta). ECMO complications collected included circuit problems, oxygenation failure, pump malfunction, clots or air in the circuit, and cannula problems. The data source of these parameters was the perfusion record.

Postprocedural complications were determined by reviewing the electronic medical records. Definitions of ECMO complications in this study followed the guidelines of the Extracorporeal Life Support Organization, available at the website https://www.elso.org/Resources/Guidelines.aspx . Bleeding complications included gastrointestinal bleeding, surgical site bleeding, hemolytic events (plasma hemoglobin> 50 mg/dL), and disseminated intravascular coagulation. Neurological complications included clinical determination of brain death, clinical and electroencephalogram determination of seizure, and computed tomography or cerebral ultrasonography evidence of central nervous system (CNS) infarction and CNS hemorrhage. Renal complications included acute kidney injury (serum creatinine >1.5 mg/dL) and/or requirement for renal replacement therapy. Cardiovascular complications included the requirement for inotropic drugs during ECMO, cardiac arrest necessitating cardiopulmonary resuscitation, and cardiac arrhythmia and myocardial stunning defined by echocardiography. In contrast, pulmonary complications included pneumothorax and pulmonary hemorrhage. Additionally, metabolic complications were defined when blood pH <7.2 or >7.6, blood glucose <40 mg/dL or >240 mg/dL, or hyperbilirubinemia was presented (direct bilirubin> 2 mg/dL, indirect bilirubin> 13 mg/dL, or total bilirubin> 15 mg/dL). Other complications of interest included limb ischemia and infectious complications, which used definitions of the U.S. Center for Disease Control definition of nosocomial infection.

Survival outcomes were recorded as in-hospital mortality or survival-to-discharge and followed the patient until 4 weeks post-procedure.

### Data Management and Statistical Methods

Data were collected using the Epidata program and analyzed with the R-program or Stata (Version 13.0). As the study design of the present study was a retrospective cohort study, missing data of non-outcome variables were found in 5% of the participants in the present cohort. Before carrying out the statistical analysis, a listwise data deletion procedure was performed for the purpose of missing value management. For description, continuous variables were reported as mean and standard deviation or median and interquartile range (IQR), while categorial data were reported as frequencies with percentages. Comparisons of continuous data between the outcome groups used the Student's *t* -test or Mann-Whitney U test, as appropriate according to the distribution type. The comparison of categorial data used Chi-square or Fisher exact tests, as appropriate. All tests were two-tailed and a *p* -value of <0.05 was considered statistically significant.

## Results

**Fig. 1:**
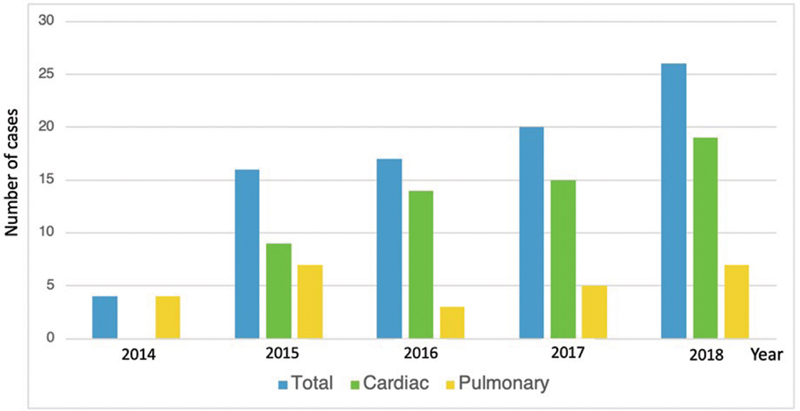
The total number of patients in which extracorporeal membrane oxygenation was initiated in each year, and their indications, including either refractory cardiac failure (cardiac) or refractory respiratory failure (pulmonary).

During the study period, 83 patients (55 males and 28 females) received ECMO support in our hospital, and the number of cases increased from four patients in the year 2014 to 26 patients in the year 2018 ( Fig. 1 ). The majority of ECMO indications belonged to refractory cardiac failure in 57 patients (68.7%), while respiratory failure was an indication in 26 patients (31.3%). Moreover, with the uptrend in ECMO employment, the cardiac indication increased. Regarding the objectives of ECMO support, 73 cases (88.0%) used the procedure with a “bridge to recovery” purpose, while eight (9.6%) were “bridge to surgery” and two (2.4%) were “bridge to transplant.” Of the 83 cases, 35 (42.2%) recovered from their primary conditions and ECMO could be weaned off, 22 (26.5%) died during the treatment, and the decision was made to withdraw ECMO in 26 (31.3%) cases. Moreover, of the 35 cases who could be weaned off from ECMO during therapy, 32 cases survived until their discharge from the hospital.

### Pre-therapeutic Conditions

The age of the patients ranged from 1 month to 83 years, with a median age of 35 years (IQR: 10, 58 years). The median age in those who survived to discharge was significantly less than those who died before discharge ( Table 1 ). Regarding primary conditions, significantly higher survival was observed in the group with ECMO for respiratory failure (57.7%), compared to those used in cardiac indications (29.9%) ( *p* -value = 0.02). Moreover, median serum pH before ECMO was 7.2 (IQR: 7.1, 7.3), while median PaO _2_ was 68.7 mm Hg (IQR: 48.9, 122.0 mm Hg), and median PaCO _2_ was 45.8 (IQR: 35.2, 57.5 mm Hg). There were no significant differences in blood gases and serum lactate parameters between the two outcome groups ( Table 1 ).

**Table 1:** Pre-ECMO parameters and comparison between those who survived and died before discharge

Parameters	All ( *N* = 83)	Survived to discharge ( *N* = 32)	Death ( *N* = 51)	*p* -Value
Age (years)	35 (11, 58)	20 (3, 54)	45 (28, 62)	<0.01
*Main indication of ECMO*				0.03
Cardiac failure	57 (68.7%)	17 (29.8%)	40 (70.2%)	
Respiratory failure	26 (31.3%)	15 (57.7%)	11 (42.3%)	
*Main purpose for ECMO*				0.07
Bridge to recovery	73 (88.0%)	29 (39.7%)	44 (60.3%)	
Bridge to cardiac surgery	8 (9.6%)	1 (12.5%)	7 (87.5%)	
Bridge to transplantation	2 (2.4%)	2 (100.0%)	0 (0.0%)	
*Pre-ECMO blood gases*				
Serum pH	7.2 (7.1, 7.3)	7.2 (7.1, 7.3)	7.2 (7.1, 7.3)	0.99
PaO _2_ (mm Hg)	68.7 (48.9, 122.0)	69.7 (49.1, 99.6)	68.7 (49.2, 125.3)	0.81
PaCO _2_ (mm Hg)	45.8 (35.2, 57.5)	46.6 (35.1, 62.2)	45.0 (35.4, 53.2)	0.73
Arterial HCO _3_ (mmol/L)	18.0 (13.9, 21.8)	18.0 (16.5, 21.5)	17.2 (13.1, 21.5)	0.34
Serum lactate (mmol/L)	14.0 (7.0, 19.5)	8.0 (2.0, 14.0)	15.5 (11.2, 20.2)	0.22

Abbreviation: ECMO, extracorporeal membrane oxygenation.

Note: All continuous parameters are presented as median (interquartile range), and categorial data are reported as frequencies with percentages.

Regarding the management of cardiopulmonary failure before ECMO support, 40 cases (48.2%) were receiving ventilatory support and 27 cases (32.5%) had prior cardiac arrest, of which seven underwent ECPR. Additionally, 11 cases (13.3%) had previously undergone cardiopulmonary bypass in the same admission and 10 cases (12.0%) had an intra-aortic balloon pump. Forty cases (48.2%) received at least one vasoactive drug before the ECMO decision. Moreover, corticosteroids were used in 12 cases (14.5%). Regarding preexisting infections, 28 cases (33.7%) were clinically diagnosed with infectious conditions, of which 24 were culture positive, including 17 with positive blood culture and 20 with positive body fluid/pus culture. Additionally, 5 of 83 cases (6.0%) had been treated with immunosuppressive agents.

### Conditions during ECMO Support

ECMO circuit implantation took place in the operating room in 78 cases (94.0%), at the intensive care ward in two cases (2.4%), and at other places—including the emergency room and cardiac catheterization center—in three cases (3.6%). ECMO types included VV-ECMO in 49 cases (59.0%), all of which used peripheral venous cannulation, and VA-ECMO in 34 cases (41.0%). Heparin was used as an anticoagulant, with the goal of achieving an activated partial activated thromboplastin time (aPTT) ratio of 150 to 220 seconds.

**Fig. 2:**
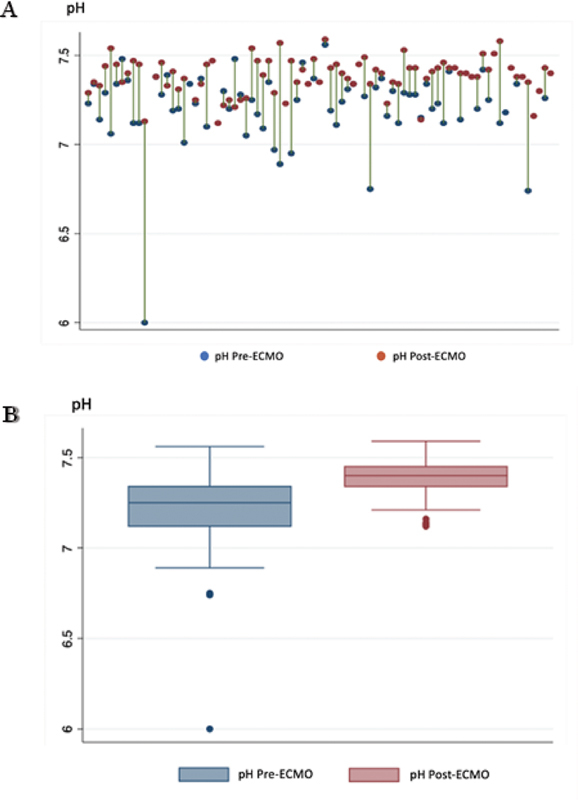
Change in blood pH after 4 hours of ECMO therapy. ( **A** ) Spike plots showing improvement in blood pH in each individual case from pre-ECMO ( *blue dots* ) to during ECMO ( *red dots* ). ( **B** ) Box plots of median blood pH pre-and during ECMO support. ECMO, extracorporeal membrane oxygenation.

At 4 to 6 hours after ECMO initiation, the median blood pH was 7.4 (IQR: 7.3, 7.5) ( Fig. 2 ), while the medians of PaO2 and PaCO _2_ were 156 mm Hg (IQR: 96.3, 281.0 mm Hg) and 36.9 mm Hg (IQR: 31.8, 42.8), respectively. Additionally, median serum bicarbonate was 23.5 mmol/L (IQR: 20.3, 27.5 mmol/L). The median flow rates at 4 and 24 hours were equal, at 90 mL/kg/min, and the median flow rate at 4 hours of ECMO support in the non-survival group (100 mL/kg/min) was significantly higher than that of the survival group (80 mL/kg/min) ( *p* -value = 0.01). The median activated clotting time (ACT) during ECMO was 172.0 seconds (IQR: 143.5, 222.0 seconds), and the median ACT in the non-survivors (181.5 seconds) was significantly higher than that of the survivors (151.5 seconds) ( Table 2 ).

**Table 2:** Patient parameters during ECMO therapy and comparisons between those who survived and died before discharge

Parameters	All ( *N* = 83)	Survived to discharge ( *N* = 32)	Death ( *N* = 51)	*p* -Value
*ECMO type*				
VA-ECMO	34 (41.0%)	16 (47.1%)	18 (52.9%)	0.19
VV-ECMO	49 (59.0%)	16 (32.7%)	33 (67.4%)	
*During ECMO blood gases* a				
Serum pH	7.4 (7.3, 7.5)	7.4 (7.3, 7.5)	7.4 (7.3, 7.5)	0.31
PaO _2_ (mm Hg)	156.0 (96.3, 281.0)	118.0 (78.8, 210.0)	181.5 (109.4, 333.8)	0.03
PaCO _2_ (mm Hg)	36.9 (31.8, 42.8)	37.2 (32.8, 42.4)	36.5 (31.3, 42.9)	0.62
Arterial HCO _3_ (mmol/L)	23.5 (20.3, 27.5)	24.2 (21.4, 28.8)	23.2 (18.5, 26.6)	0.31
Flow rate at the fourth hour (mL/kg/min)	90 (75, 100)	80 (70, 100)	100 (80, 100)	0.01
Flow rate at the 24th hour (mL/kg/min)	90 (75, 100)	83 (74, 100)	90 (75, 100)	0.08
Activated clotting time (s)	172 (144, 222)	152 (140, 184)	182 (156, 231)	<0.01

Abbreviations: ECMO, extracorporeal membrane oxygenation; VA, venoarterial; VV, venovenous.

a Arterial blood gases and activated clotting time were taken at the fourth hour after ECMO initiation.

During ECMO support, adjunctive use of vasoactive drugs was necessary in 78 cases (94.0%) and ventilator support was continued in 82 cases (98.8%). Moreover, upon serial evaluation of ACT, 78 cases had a value out of the expected range of 140 to 180 seconds. Recorded metabolic derangement included hyperbilirubinemia in 18 cases (21.7%), metabolic acidosis in six cases (7.2%), and one case each of hypo- and hyperglycemia. Limb complications occurred in three patients and one required a fasciotomy. Bleeding and hematologic complications occurred in 38 patients (45.8%), while other complications included seizures, acute kidney injury, new episodes of infection, and cardiac tamponade ( Table 3 ).

**Table 3:** Complications encountered during ECMO therapy in this study

Complications	Frequency (%)	Mortality
*Circuit and tubing problems*	9 (10.8%)	9/9 (100%)
Oxygenation failure	4 (4.8%)	
Intra-circuit clot	4 (4.8%)	
Cannula problem	1 (1.2%)	
*Hematologic complications*	38 (45.8%)	26/38 (68.4%)
Gastrointestinal hemorrhage	5 (6.0%)	
Cannula site bleeding	24 (28.9%)	
Surgical site bleeding	20 (24.1%)	
Hemolysis	3 (3.6%)	
Disseminated intravascular coagulation	3 (3.6%)	
*Metabolic complications*	26 (31.33%)	17/26 (65.4%)
Hypoglycemia (serum glucose <40 mg/dL)	1 (1.2%)	
Hyperglycemia (serum glucose >240 mg/dL)	1 (1.2%)	
Metabolic acidosis (pH <7.0)	6 (7.2%)	
Hyperbilirubinemia	18 (21.7%)	
*Neurological complications*	12 (14.5%)	8/12 (66/7%)
Brain death	5 (6.0%)	
Seizure (clinical diagnosis)	6 (7.2%)	
Seizure (EEG)	1 (1.2%)	
Intracranial hemorrhage	2 (2.4%)	
*Renal complications*	45 (54.22%)	31/45 (68.9%)
Acute kidney injury: creatinine 1.5–3.0	24 (28.9%)	
Acute kidney injury: creatinine >3.0	5 (6.0%)	
Hemodialysis requirement	16 (19.3%)	
CVVH requirement	18 (21.7%)	
*Cardiac complications*	71 (85.5%)	44/71 (62.0%)
Inotropic drug requirement	71 (85.5%)	
Cardiopulmonary resuscitation	8 (9.6%)	
Myocardial stunning	15 (18.1%)	
Cardiac arrhythmia	29 (34.9%)	
Hypertension requiring vasodilators	2 (2.4%)	
PDA reopen (left to right shunt)	1 (1.2%)	
Cardiac tamponade	6 (7.2%)	
*Pulmonary complications*	8 (9.6%)	7/8 (87.5%)
Pneumothorax	5 (6.0%)	
Pulmonary hemorrhage	3 (3.6%)	
*Infectious complications*		2/6 (33.3%)
Newly culture-positive infection	6 (7.2%)	
*Vascular complication*		2/3 (66.7%)
Limb ischemia	3 (3.61)	

Abbreviations: CVVH, continuous venovenous hemofiltration; ECMO, extracorporeal membrane oxygenation; EEG, electroencephalogram; PDA, patent ductus arteriosus.

One or more circuit problems were reported in nine patients (10.8%). Problems included oxygenator malfunction (four cases), intra-circuit clots (four cases), and a case which experienced a cannula problem.

### ECMO Outcomes and Postprocedural Complications

Of the 83 patients, 35 (42.2%) survived until ECMO support could be weaned off, while 22 cases (26.5%) died during the treatment and 26 cases (31.3%) prematurely withdrew the support. Reasons for withdrawal included family decision in 20 cases, multiorgan failure in four cases, and uncontrolled hemorrhagic complication in two cases. Average ECMO duration in all cases was 7.5 days (range, 8 hours to 41 days), 8.9 days in the survival-from-ECMO cases, 5.2 days in those who died during the treatment, and 7.4 days in those who withdrew. Duration of ECMO in cases who survived to discharge (9.7 days) was significantly longer than those who had in-hospital mortality (6.15 days, *p* -value = 0.03).

**Fig. 3:**
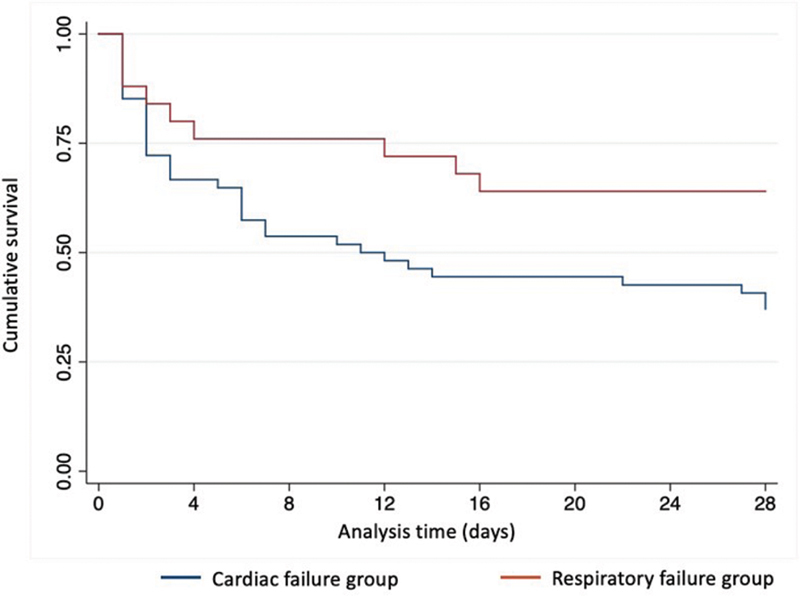
Survival probability of extracorporeal membrane oxygenation, compared between clinical indications (Log-rank *p* -value = 0.02).

On follow-up until 4 weeks after weaning-off from ECMO, 6 of 35 cases had postprocedural complications, including two infectious complications and one each of the CNS, pulmonary, hepatic, and other organs. On survival analysis, patients who used ECMO due to respiratory failure had significantly better survival probability when compared to the cardiac failure group ( Fig. 3 ).

### Factors Associated with Survival Outcomes in ECMO Patients

On summary of the univariate analyses, factors associated with higher in-hospital mortality in our ECMO patients included older age of the patients, cardiac indication of ECMO, higher flow rate at the fourth hour of therapy, higher ACT, and higher numbers of complications during therapy ( Table 4 ). On multivariate analysis using stepwise multiple logistic regression analysis, only the number of complications (OR, 1.46; 95% CI, 1.13–1.91), age (OR, 1.02; 95% CI, 1.01–1.05), and ACT at the fourth ECMO hour (OR, 1.01; 95% CI, 1.00–1.02) were significantly associated with the in-hospital mortality in the final model. Further analysis found that ACT> 140 seconds at the fourth hour of ECMO was associated with higher in-hospital mortality (OR, 3.22; 95% CI, 1.09–9.49).

**Table 4:** Univariate logistic regression analysis showing factors associating with in-hospital mortality in ECMO patients

Parameters	OR	95% confidence interval	*p* -Value
Older age	1.02	1.01–1.05	0.004
Cardiac failure as ECMO indication	3.21	1.22–8.40	0.018
Increased flow rate at fourth ECMO hour	1.04	1.01–1.07	0.017
Increased activated clotting time	1.01	1.00–1.02	0.014
Increased number of complications	1.32	1.04–1.68	0.021

Abbreviations: ECMO, extracorporeal membrane oxygenation; OR, odds ratio.

## Discussion

ECMO technology itself is not a definitive therapy, but an artificial organ system that provides extended cardiopulmonary support for patients with refractory cardiac or respiratory failure until their own physiology has adequately recovered, either spontaneously or by other specific treatments. During the first 5 years of adopting this technology, ECMO bridged 39% of our patients to their survival until discharge, with 58% of these being for respiratory failure and 30% for cardiac indications. Fig. 3 shows that survival in respiratory failure cases was significantly better, which was consistent with other series. 13 According to the Extracorporeal Life Support Organization (ELSO) registry, reported as of 2018, 59% of 16,337 registered ECMO cases with respiratory failure and 42% of 15,942 cardiac cases survived to discharge. 14 While survival in our respiratory cases was comparable with that of the ELSO registry, lower survival in cardiac cases suggested that we need to improve the care process in ECMO for cardiac patients, including case selection. We also found that a higher number of ECMO complications were independently associated with higher mortality.

Of our 83 patients, 81 experienced at least one complication during their ECMO therapy and we found that more complications significantly jeopardized survival. Most of the common complications our team encountered were cardiac, renal, and hematologic complications. Moreover, among hematologic complications, bleeding was the most frequent and probably the most important complication. 13 A recent study by Aubron et al focusing on bleeding complications in ECMO patients found that higher aPTT was an independent factor for bleeding in ECMO patients. 15 In our patients, 94% were reported to have out-of-range ACT and increased ACT level was one factor independently associated with mortality. Therefore, more stringent control of ACT should be our strategy to reduce bleeding complications, thereby improving survival outcomes.

Age was another factor that was found to be associated with survival. This may be explained by the fact that the physiologic reserve of younger patients was higher. However, we also found a higher ECMO withdrawal rate in cases aged >60 years (42.1%), compared to younger patients (28.12%). Circuit problems were reported by the perfusionists in 11% of the patients and all the patients with these events died. These data emphasized the importance of regular system maintenance and personnel training. Furthermore, neurological complications occurred in 14.5% of our cases and the most common problem was convulsion, followed by brain death. Causes of neurological complications in patients undergoing ECMO therapy can be both pre-ECMO conditions that result in poor perfusion and during ECMO support when coagulation derangement may occur, including either hypercoagulability or coagulopathy. Therefore, careful monitoring of coagulation status during this time is crucial. 16 In addition to coagulation-related problems, perfusion-related complications such as limb ischemia and acute kidney injury (AKI) also occurred in our patients. In a study focusing on renal issues in ECMO patients, 42% of their 196 cases required renal replacement therapy during ECMO. Their study found that serum lactate was a strong predictor of AKI patients requiring renal replacement therapy. Although the number of cases with serum lactate in our series was not enough to analyze for this association, we found a trend toward a higher incidence of renal complications in our cases with pre-ECMO lactate >4 mmol/L (10/12), compared to those with normal lactate levels (1/3).

The limitation of this study was in its retrospective nature, in which there were missing data and under-recorded events. However, the strength of the study lies in the fact that the data was taken during and after ECMO and were from structured record forms used by perfusionists and the surgical team.

In summary, the study appraised an early experience of ECMO practice in a university hospital in southern Thailand. The study found a general survival-to-discharge rate of 39%. Additionally, the modifiable factors that were independently associated with survival included the number of complications and ACT. Therefore, to improve the overall outcomes, a strategy should be put into place to reduce complications during ECMO support.
